# On a Simple Mode of Reducing a Dislocated Elbow

**Published:** 1857-06

**Authors:** 


### *On a Simple Mode of Reducing a Dislocated Elbow.* By M. Bidard, of Arras.

In a recent communication to the Sociéte de Chirurgie de Paris, M. Bidard relates a case in which a dislocated elbow was reduced in a very simple manner, after the ordinary means had failed. A child, aged 13, had dislocated his elbow, and the dislocation had been reduced in the ordinary way. A month later the elbow was again dislocated. On this occasion the child said nothing about the accident, and five weeks passed before the mischief was discovered, and the attempts at reduction repeated. These attempts failed. It then occurred to Mr. Bidard to persuade the child to swing himself by both hands from a cross beam of wood, and to allow his hands to be held in this position by another person when he became tired. These swingings were continued for fifteen or twenty minutes at a time, and repeated every morning and evening; and the result was that the displacement had entirely disappeared on the seventh day. It appears from the account, that the displacement diminished progressing between the first and ninth suspension; and that the rest of the deformity disappeared suddenly during the fourteenth suspension.

This method, as Mr. Larrey observed afterwards, possesses some analogy to that of the *door*, as formerly practiced by some surgeons, but with this difference, that the reduction is effected gradually in this case and suddenly in that.

As to the rest we are disposed to think there need have been no difficulty if the chloroform had been employed; for, unquestionably, dislocations of much older standing are easily reducible with the help of this agent.—*Gaz. Hebdom. de Mét. et Chir.*

---

### *On Œdema Glottidis, resulting from Typhus Fever.* By Thomas Addis Emmet, M. D. (American Journal of the Medical Sciences, July, 1856.

Dr. Emmet draws attention to two forms in which œdema glottidis occurs as a secondary affection of typhus, either as a result of a reactive ulceration of the mucous membrane of the air passages, in consequence of typhous deposit, or as a result of the debilitated condition of the patient alone remaining after the subsidence of the primary disease; in the former variety the infiltration takes place slowly; in the latter, with great rapidity, so as to cause almost instantaneous death. Dr. Emmet supports his views by cases. He is of opinion that œdema glottidis is more frequently the cause of the fatal issue of diseases than is commonly supposed. With regard to the performance of tracheotomy or laryngotomy, it is not advisable at all in the cases of laryngo-typhus, since in every fatal case of this affection, bronchitis was found to co-exist; a more favorable issue may be expected to follow where it is performed for simple œdema. The relative frequency of œdema of the glottis as a sequel of typhus, may be gathered from the fact, that out of 1931 cases of typhus, 23 presented the laryngo-typhous, 7 the simple form of œdema glottidis.

